# Association between Urinary Calcium Excretion and Estimated Glomerular Filtration Rate Decline in Patients with Type 2 Diabetes Mellitus: A Retrospective Single-center Observational Study

**DOI:** 10.3390/jcm7070171

**Published:** 2018-07-10

**Authors:** Hodaka Yamada, Shunsuke Funazaki, Daisuke Suzuki, Rika Saikawa, Masashi Yoshida, Masafumi Kakei, San-e Ishikawa, Yoshiyuki Morisita, Kazuo Hara

**Affiliations:** 1 Department of Medicine, Division of Endocrinology and Metabolism, Jichi Medical University Saitama Medical Center, 1-847 Amanuma-cho, Omiya-ku, Saitama 330-8503, Japan; s.funazaki@jichi.ac.jp (S.F.); middleroad0619@jichi.ac.jp (D.S.); rika.s@jichi.ac.jp (R.S.); myoshida-md@jichi.ac.jp (M.Y.); mkakei@jichi.ac.jp (M.K.); hara@jichi.ac.jp (K.H.); 2 Division of Endocrinology and Metabolism, International University of Health and Welfare Hospital, 537-3 Iguchi, Nasushiobara, Tochigi 329-2763, Japan; saneiskw@jichi.ac.jp; 3 Department of Medicine, Division of Nephrology, Jichi Medical University Saitama Medical Center, 1-847 Amanuma-cho, Omiya-ku, Saitama 330-8503, Japan; ymori@jichi.ac.jp

**Keywords:** Urinary calcium excretion, chronic kidney disease, diabetes, fibroblast growth factor 23, mineral and bone disorder

## Abstract

Urinary calcium excretion is not known to predict progression of renal dysfunction in patients with type 2 diabetes mellitus. This study aimed to investigate associations between urinary calcium excretion and progression of estimated glomerular filtration rate (eGFR) in type 2 diabetic patients. This study was a retrospective, single-center, observational cohort study. We enrolled a total of 89 patients with type 2 diabetes mellitus and the average follow-up period was 7.2 ± 1.0 years. We divided patients into two groups based on the median of annual decline in the slope of eGFR, then defined the over-median population as the progressed group and under-median population as the non-progressed group. Median of annual decline in the slope of eGFR was −1.1 mL/min/1.73 m^2^/year. Correlation coefficient analysis showed positive correlation of urinary calcium excretion with eGFR (*r* = 0.39, *p* < 0.001). Multivariate logistic analysis showed that baseline eGFR and urinary calcium excretion were independent variables for progression of eGFR decline. Urinary calcium excretion could be a useful metabolic parameter for predicting decline in slope of eGFR in patients with type 2 diabetes mellitus.

## 1. Introduction

Chronic kidney disease (CKD) is an important risk factor for cardiovascular complications negatively affecting quality of life significantly [1]. In particular, diabetes mellitus is a factor for CKD progression [2,3]. Preventing progression of renal impairment is important from the perspective of preventing hemodialysis and its related national health care costs. Over the last decade, CKD-related mineral and bone disorder (CKD-MBD) involving fibroblast growth factor 23 (FGF23), and calcium and phosphate metabolism, has attracted attention as a new risk factor for progression of atherosclerosis and CKD [4]. In early stage CKD, elevated FGF23 maintains serum phosphate concentration. However, in the progressed stage, FGF23 cannot excrete enough phosphate, thus causing hyperphosphatemia that further progresses to vascular calcification. Renal Function and injury are well known poor prognostic factors [4]. Notably, urinary albumin excretion is an established independent risk factor for progression of renal impairment in patients with type 2 diabetes. However, the contribution of urinary electrolytes in the progression of CKD in patients with type 2 diabetes mellitus is still unclear. Urinary potassium excretion was decreased in progressed CKD [5], and higher urinary potassium excretion was associated with slow decline of renal function and lower risk of cardiovascular events in patients with type 2 diabetes [6]. Contrarily, only few studies have examined the relationship between urinary calcium excretion and CKD progression. Hypercalciuria is considered a clinical feature in early diabetes and was reduced after insulin therapy [7]. Capacity of urinary calcium excretion is decreased in diabetic patients with renal impairment [8]. There is no evidence that urinary calcium excretion can predict progression of renal dysfunction in patients with type 2 diabetes mellitus. Here, we investigated the relationship between urinary calcium excretion and estimated glomerular filtration rate (eGFR) progression in type 2 diabetic patients.

## 2. Materials and Methods

### 2.1. Study Participants

This was a retrospective, single-center, observational cohort study. Study participants were enrolled based on our previous study cohort between March 2005 and January 2007 [9]. Briefly, subjects were hospitalized at the Jichi Medical University Saitama Medical Center for 2 weeks to learn how and why they should practically control their blood glucose. Daily calcium and phosphate intake were managed between 600 and 700 mg/day and 1000 and 1100 mg/day by a nutritionist, respectively. The following inclusion criteria were considered for enrollment in this study: (1) patients aged >20 years and having type 2 diabetes and (2) patients who visited the outpatient clinic of our hospital for over 1 year after discharge. Additionally, the following criteria were considered for exclusion from the study: (1) diabetic ketoacidosis/ketosis or hyperosmolar hyperglycemic syndrome, (2) infectious diseases, (3) any malignancy during treatment, (4) existing pregnancy, and (5) patients taking supplemental vitamin D, calcium carbonate, or phosphate binders.

### 2.2. Data Collection

Demographic data of patients, such as age, sex, body mass index (BMI), blood pressure, and duration of diabetes mellitus, were obtained. Twenty-four-hour urine collection was performed during hospitalization. Serum intact FGF23 was measured using a commercial enzyme-linked immunosorbent assay (ELISA) kit (Kainos, Tokyo, Japan). After discharge, we followed eGFR of patients who visited our hospital during 2016. If patients visited another hospital, we used their clinical laboratory data at their final visit. We divided patients into two groups based on the median of annual decline in the slope of eGFR (mL/min/1.73 m^2^/year), then defined the over-median population as the progressed group and the under-median population as the non-progressed group. Renal function was determined with calculation of eGFR assessed using the modification of diet in renal disease equation revised for the Japanese population by the Japanese Society of Nephrology as follows: eGFR (mL/min/1.73 m^2^) = 194 × serum creatinine (mg/dL)^−1.094^ × age^−0.287^ × 0.739 (if female). Corrected serum calcium levels were calculated using the following equation: serum calcium + (4-serum albumin) (if serum albumin was ≤4 g/dL).

### 2.3. Ethical Approval

This study was approved by the Ethics Committee of the Jichi Medical University, Saitama Medical Center (No. S17-007) and was performed in compliance with the Declaration of Helsinki. Formal consent is not required for this study type.

### 2.4. Statistical Analysis

Data are expressed as means ± standard deviation (SD), and skewed variables are described as medians with an interquartile range. Baseline characteristics between the two groups were compared using the Mann-Whitney *U* test or Student’s *t*-test. Categorical variables were compared using Fisher’s exact test. Pearson’s or Spearman’s correlation coefficient analysis were used for linear correlations between urinary calcium excretion and serum calcium, phosphate, FGF23 or baseline eGFR. To examine the effects of various clinical factors on the deterioration of renal decline speed, the following well known risk factors were selected for multivariate logistic analysis: age, sex, body mass index (BMI), duration of diabetes, hypertension, smoking status, glycated hemoglobin (HbA1c), urinary albumin excretion, baseline eGFR expected for our factor of interest, and urinary calcium excretion. These continuous variables were divided into two groups based on their median in multivariate analysis. Serum intact FGF23 (ng/L), urinary albumin excretion (mg/day), and urinary calcium excretion (mg/dL) were transformed into logarithmic variables before multivariate logistic analysis because these parameters revealed skewed distribution. There were no missing data in any variable. All analyses were performed using EZR (Jichi Medical University Saitama Medical Center), a graphical user interface for R (The R Foundation for Statistical Computing, ver. 2.13.0) and a modified version of the R commander (ver. 1.6-3) designed to add statistical functions frequently used in biostatistics [10]. A value of *p* < 0.05 was considered significant.

## 3. Results

**Figure 1:**
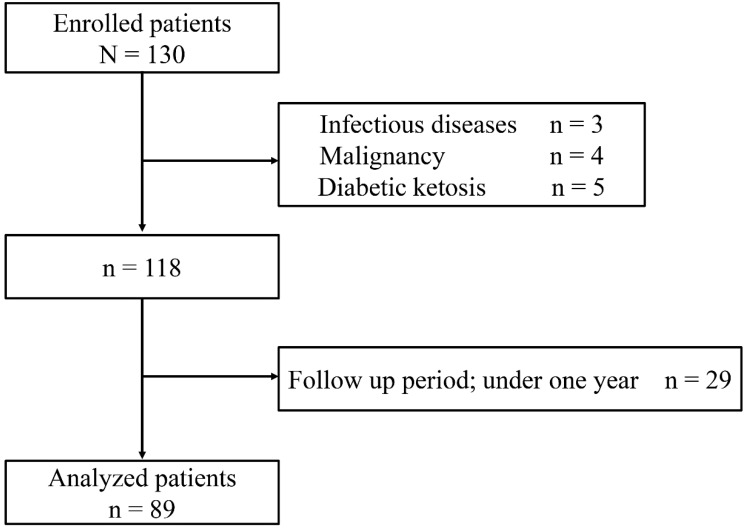
Flowchart of patient selection.

**Figure 2:**
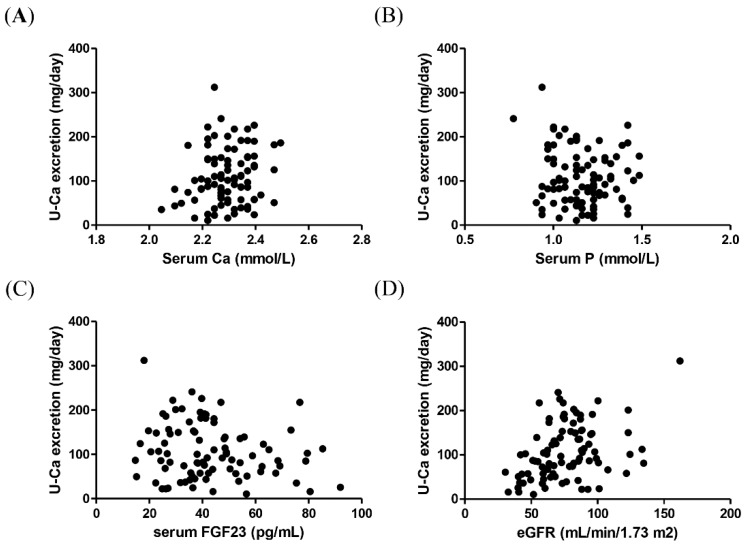
Correlation between Correlation of urinary calcium excretion (U-Ca) and baseline mineral and renal parameters (overall, *n* = 89). Correlation of urinary calcium excretion (U-Ca) with serum calcium (Ca) (*r* = 0.18, *p* = 0.08) (**A**), serum phosphate (P) (*r* = −0.11, *p* = 0.30) (**B**), serum intact fibroblast growth factor 23 (FGF23) (*r* = −0.13, *p* = 0.23) (**C**), and estimated glomerular filtration rate (eGFR) (*r* = 0.39, *p* < 0.001) (**D**) in patients with type 2 diabetes.

Initially, 130 eligible patients were identified; however, 3 of those were excluded for infectious diseases, 4 for malignancy, and 5 for diabetic ketosis. Of the remaining 118 patients, 29 were excluded owing to their less than one-year follow-up period (Figure 1). Finally, a total of 89 patients with type 2 diabetes mellitus were enrolled, and the average follow-up period was 7.2 years. Their basic characteristics are shown in Table 1. Median of annual decline in the slope of eGFR was −1.1 mL/min/1.73 m^2^/year. The progressed group was older and had a longer history of diabetes mellitus than the non-progressed group. In addition, eGFR was significantly decreased in the progressed group compared with the non-progressed group. Regarding mineral metabolic parameters, urinary calcium excretion was lower, whereas serum FGF23 levels were higher in the progressed group. There were no differences in serum calcium or serum phosphate. Overall, correlation coefficient analysis showed a positive correlation of urinary calcium excretion with baseline eGFR (*r* = 0.39, *p* < 0.001), but not with serum calcium (*r* = 0.18, *p* = 0.09), serum phosphate (*r* = −0.11, *p* = 0.30), or serum FGF23 (*r* = −0.12, *p* = 0.26) (Figure 2). To analyze significant variables associated with the decline of eGFR, we performed multiple logistic model analysis (Table 2). In the multivariate logistic analysis, baseline eGFR and urinary calcium excretion (logarithmic transformed) showed an association with the eGFR decline.

## 4. Discussion

In the present study, we found that urinary calcium excretion decreased significantly in the progressed group compared with the non-progressed group. In addition, urinary calcium excretion associated with the slope of eGFR declined in type 2 diabetic patients. These results suggested that urinary calcium excretion can predict progression of eGFR decline.

A recent population-based study revealed that urinary calcium excretion was correlated with eGFR in both men and women, with serum calcium in women, and with vitamin 25(OH)D_3_ [11]. In early-stage CKD, urinary excretion of calcium is decreased [12]. Our study including type 2 diabetic patients showed no relationship between urinary calcium excretion and serum calcium, but urinary calcium excretion had a significant correlation with eGFR. This difference may be attributed to a difference in the study population. It was reported that urinary calcium excretion was decreased and serum FGF23 was elevated, even if eGFR was preserved, in a diabetic population compared with a non-diabetic population [8].

Approximately 98% of glomerulus-filtered calcium is reabsorbed along the nephron, mainly in the proximal tubule (approximately 65%) [13]. Several studies have revealed that diabetic patients have tubular injury, and the pathophysiological findings could be seen before occurrence of albuminuria [14,15]. Indeed, urinary kidney injury molecule-1 (KIM-1), a specific proximal tubule injury marker, was elevated in early-stage CKD in normoalbuminuric patients with type 2 diabetes mellitus [16]. Moreover, a recent case-control study using the Action to Control Cardiovascular Risk in Diabetes showed that plasma KIM-1 concentration was a risk marker for higher decline in eGFR in early diabetic kidney disease [17]. These results suggest that proximal tubule injury occurred in diabetic patients with early-stage CKD. We may consider that urinary calcium excretion capacity reflects subclinical proximal tubular damage indirectly and predicts future progression of renal impairment. We also found a correlation between correlation of urinary calcium excretion (U-Ca) and decline in the slope of eGFR in all patients (*p* = 0.05, *r* = 0.200; Supplemental Figure S1). However, pathological association between urinary calcium excretion and decline in eGFR slope was unclear, and further study is needed to elucidate the exact mechanism.

Reduced baseline eGFR and elevated FGF23 level are reported to predict progression of eGFR decline in diabetic patients [18,19,20,21]. Our result was not consistent with these previous studies. Serum FGF23, a bone-derived phosphaturic hormone, is an early serological biomarker in patients with CKD, and in more progressed CKD stages, urinary phosphate excretion is decreased [22]. Recently, Dhayat et al. reported that daily urine calcium excretion was negatively associated with circulating C-terminal FGF23 in a population-based, cross-sectional study [23]. FGF23 reabsorbs glomerulus-filtrated calcium in distal tubule via transient receptor potential vanilloid 5 (TRPV5), a calcium-permeable ion channel [24]. In our study, we found no relationship between serum FGF23 and urinary calcium excretion. We measured full-length FGF23, but C-terminal FGF23 was measured by ELISA in the previous study. In addition, our study population was composed of all type 2 diabetic patients. These differences in the study population and assay method may be responsible for the conflicting results. It was shown that lower soluble klotho level was independently associated with a higher risk of rapid kidney function decline [25]. α-Klotho is known as a co-receptor for FGF-23, a putative aging suppressor, and activates TRPV5 leading to calcium reabsorption in the distal tubule [26]. On the other hand, FGF23 also promotes calcium reabsorption. Taken together, elevation of serum FGF23 and reduction in soluble klotho occur in early-stage CKD in diabetic patients. Otherwise, in the CKD state, the parathyroid hormone (PTH) concentration is elevated. PHT increased TRPV5 mRNA in the kidney [27]. PTH might affect calcium reabsorption via TRPV5. From this study, we infer that the urinary calcium excretion capacity reflects the residual renal nephron. However, the underlying mechanism is unknown. There are few clinical studies that focused on or examined an association between urinary calcium excretion and the progression of renal impairment. Further studies will help clarify the mechanism through which lower urinary calcium was associated with the risk for renal impairment progression.

There were limitations in the present study. First, this was a retrospective single-center study, but not a prospective protocol. We could not evaluate how urinary calcium excretion is involved in eGFR decline. Second, we could examine a limited number of variables in logistic multivariate analysis because of the small number of subjects. In particular, we could not adjust for bone formation (osteoporosis, fracture, and bone density), which is decreased in diabetic patients [28], PTH, soluble Klotho, and vitamin D status. In addition, our study included all hospitalized patients. Finally, the enrolled participants were all hospitalized for uncontrollable hyperglycemia. Comparison with other renal diseases such as primary glomerulonephritis and polycystic kidney disease is needed. A larger sample size with a prospective design are necessary.

## 5. Conclusions

In summary, urinary calcium excretion can be a useful metabolic parameter for predicting decline in the slope of eGFR in patients with type 2 diabetes mellitus. These findings suggest a relationship between urinary mineral metabolic factors and eGFR decline in patients with type 2 diabetes mellitus.

## Figures and Tables

**Table 1:** Baseline demographic data of participants and differences between the progressed group and the non-progressed group.

Variables and Parameters	Over All (*n* = 89)	Non-Progressed (*n* = 44)	Progressed (*n* = 45)	*p* Value
Age (years)	63 ± 11	59 ± 12	67 ± 8.3	<0.001
sex, male (%)	45 (51)	26 (59)	19 (42)	0.140
BMI (kg/m^2^)	25.1 ± 4.50	25.3 ± 5.1	24.9 ± 4.0	0.747
Duration of Diabetes (years)	12 ± 8.0	10 ± 7.1	14 ± 8.3	0.022
Hypertension, *n* (%)	49 (55)	20 (45)	29 (64)	0.090
Smoking status, *n* (%)	27 (30)	14 (32)	13 (29)	0.820
HbA1c (mmol/L)	78.0 ± 16.0	78.7 ± 17.6	77.2 ± 14.4	0.661
Systolic BP (mmHg)	132 ± 19	127 ± 18	136 ± 19	0.025
Diastolic BP (mmHg)	74 ± 13	75 ± 10	74 ± 16	0.795
TC (mmol/L)	5.05 ± 1.01	5.13 ± 1.02	4.98 ± 1.01	0.464
TG (mmol/L)	2.91 (2.09–4.14)	2.90 (2.27–3.70)	3.10 (2.07–4.60)	0.733
HDL-C (mmol/L)	1.25 ± 0.42	1.30 ± 0.46	1.21 ± 0.37	0.275
Baseline eGFR (mL/min/1.73 m^2^)	74 (59–88)	72 (58–86)	80 (64–93)	0.112
Serum calcium (mmol/L)	2.29 ± 0.09	2.29 ± 0.09	2.29 ± 0.09	0.945
Serum phosphate (mmol/L)	1.16 ± 0.154	1.17 ± 0.177	1.16 ± 0.130	0.791
serum FGF23 (ng/L)	40 (31.0–52.6)	36 (27–45)	44 (36–59)	0.003
Urinary albumin excretion (mg/day)	12 (7.0–67)	10 (4.8–19)	16 (7.7–121)	0.041
Urinary calcium excretion (mg/day)	97 (58–150)	108 (72–154)	82 (51–146)	0.150
Follow-up time (years)	7.2 ± 1.0	7.3 ± 0.93	7.2 ± 1.1	0.578
eGFR after follow up (mL/min/1.73 m^2^)	62 (48–82)	76 (57–88)	54 (42–72)	<0.001
eGFR decline speed (/year)	−1.1 (−2.6–0.19)	−0.17 (−0.44–0.94)	−2.6 (−5.1–2.0)	<0.001

Data are expressed as means ± standard deviation (SD), and skewed variables are described as medians with an interquartile range. BMI: body mass index; HbA1c: glycated hemoglobin; BP: blood pressure; TC: total cholesterol; TG: triglycerides; HDL-C: high density lipoprotein cholesterol; eGFR: estimated glomerular filtration rate; FGF23: Fibroblast growth factor 23.

**Table 2:** Multivariate logistic analysis showing independent variables in progression of eGFR decline in patients with type 2 diabetes mellitus.

	OR	95% CI	*p* Value	OR	95% CI	*p* Value
Age (>62 years)	1.9	0.65–5.6	0.244			
Male	1.4	0.50–4.1	0.494			
BMI (>24.5 kg/m^2^)	0.69	0.24–2.0	0.498			
Duration of diabetes (>10.5 years)	1.4	0.49–4.2	0.521			
Hypertension	1.5	0.53–4.2	0.454			
Smoking status	1.1	0.38–3.2	0.862			
HbA1c (>76 mmol/L)	0.96	0.37–2.5	0.925			
Baseline eGFR (>77 mL/min/1.73 m^2^)	4.6	1.3–16.1	0.018	3.3	1.1–9.7	0.033
Log serum FGF23 (>1.61)	1.3	0.48–3.6	0.599			
Log Urinary albumin excretion (>1.14)	1.2	0.45–3.4	0.692			
Log Urinary calcium excretion (≤1.99)	4.3	1.5–12.2	0.005	6.1	2.1–18.0	0.001

OR: odds ratio; CI: confidence interval; Log: logarithmic transformed; BMI: body mass index; HbA1c: glycated hemoglobin; eGFR: estimated glomerular filtration rate; FGF23: fibroblast growth factor 23.

## Supplementary Materials

The following is available online at http://www.mdpi.com/2077-0383/7/7/171/s1, Figure S1: Correlation between Correlation of urinary calcium excretion (U-Ca) and the decline in the slope of eGFR in all patients (*p* = 0.05, *r* = 0.200).
