# Supplementary material for: Association between Urinary Calcium Excretion and Estimated Glomerular Filtration Rate Decline in Patients with Type 2 Diabetes Mellitus: A Retrospective Single-center Observational Study
Source: J Clin Med. 2018 Jul 10;7(7):171. doi: 10.3390/jcm7070171 (PMC6069054; doi:10.3390/jcm7070171)
Supplement: Supplementary file 1 [file jcm-07-00171-s001.pdf]

Supplementary Materials: Association between Urinary Calcium Excretion and Estimated Glomerular Filtration Rate Decline in Patients with Type 2 Diabetes Mellitus: A Retrospective Single-center Observational Study

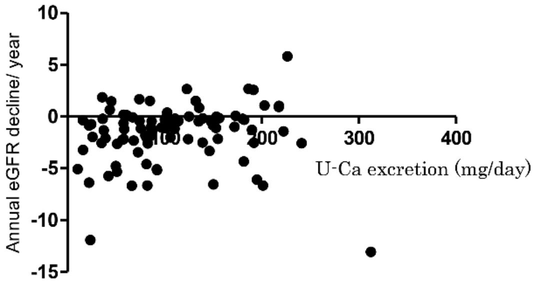

Figure S1. Correlation between Correlation of urinary calcium excretion (U-Ca) and the decline in the slope of eGFR in all patients ($p = 0.05$, $r = 0.200$).
